# Diagnostic Value of In Vitro Tests for Peanut Allergy in Children Without Clinical Exposure: A High-Specificity Rule-In Decision Pathway—Preliminary Findings from a Single-Center Study in Polish Children

**DOI:** 10.3390/children13010090

**Published:** 2026-01-07

**Authors:** Julia Tworowska, Kinga Lis, Zbigniew Bartuzi, Aneta Krogulska

**Affiliations:** 1 Department of Pediatrics, Allergology and Gastroenterology, Collegium Medicum in Bydgoszcz, Nicolaus Copernicus University in Torun, 87-100 Bydgoszcz, Poland; 2 Department of Allergology, Clinical Immunology and Internal Diseases, Collegium Medicum in Bydgoszcz, Nicolaus Copernicus University in Torun, 87-100 Bydgoszcz, Poland; kinga.lis@cm.umk.pl (K.L.); bartuzi@cm.umk.pl (Z.B.)

**Keywords:** peanut hypersensitivity, Ara h 2 allergen, basophil activation test, skin tests, child

## Abstract

**Highlights:**

**What are the main findings?**

- sIgE to Ara h 2 and peanut extract showed the highest individual diagnostic accuracy in children with absent or uncertain peanut exposure.
- A high-specificity, multistage in vitro decision pathway achieved 100% specificity, allowing the avoidance of unnecessary OFC in definitively allergic patients in over 28% of sensitized children.

**What are the implications of the main findings?**

- In selected real-life clinical scenarios, structured laboratory-based decision pathways may reduce unnecessary OFCs.
- Due to feasibility limitations, BAT should be considered a confirmatory rather than a mandatory diagnostic step.

**Abstract:**

**Background:** Diagnosing peanut allergy (PA) in children without known exposure remains challenging due to the need to distinguish true clinical allergy from asymptomatic sensitization. This study aimed to evaluate the diagnostic performance of individual and combined in vitro markers, particularly sIgE to Ara h 2, and to develop a multistage decision pathway that may reduce reliance on oral food challenge (OFC). **Methods:** Eighty children with suspected peanut allergy were prospectively enrolled. All participants, including healthy controls, underwent skin prick testing (SPT), measurement of sIgE to peanut and Ara h 2, and basophil activation testing (BAT). A multistage diagnostic algorithm incorporating these markers was constructed, and its performance was assessed using ROC analysis, predictive values, and likelihood ratios. A secondary analysis evaluated a simplified decision pathway excluding BAT. **Results:** sIgE to Ara h 2 demonstrated excellent individual performance (AUC 0.889), with 96.6% PPV at the optimal cut-off. The full multistage decision pathway (SPT + sIgE + BAT when interpretable) achieved 100% specificity and avoided OFC in 28.6% of children. However, BAT feasibility was limited; over 25% of results were uninterpretable. The simplified decision pathway (SPT + sIgE to Ara h 2) preserved 100% specificity and enabled the avoidance of OFC in 27.5% of cases, with slightly lower sensitivity. **Conclusions:** A structured in vitro diagnostic approach using sIgE to Ara h 2 and SPT can reliably identify peanut allergy in selected pediatric patients, particularly those without a reliable peanut exposure history. BAT enhances specificity but should be considered a confirmatory tool due to feasibility limitations.

## 1. Introduction

Peanut allergy (PA) is one of the most clinically significant IgE-mediated food allergies in childhood, associated with a high risk of severe reactions and a substantial impact on quality of life [1]. During the last decade, landmark trials, most notably the Learning Early About Peanut Allergy (LEAP) study and its LEAP-On extension, have redefined our understanding of sensitization, early exposure, and long-term immune tolerance [2,3]. These studies demonstrated that early and sustained peanut consumption can prevent the development of PA in high-risk infants. Their findings have influenced prevention guidelines worldwide and highlighted the complexity of distinguishing clinically relevant allergy from asymptomatic sensitization [1].

In routine clinical practice, however, a growing number of children undergo allergy testing without a clear or reliable clinical history. This is driven by rising parental anxiety, broad screening strategies, and the accessibility of component-resolved diagnostics (CRD). As a result, clinicians are frequently confronted with laboratory results, most notably serum sIgE to peanut and skin prick testing (SPT), in children who have never ingested peanut or whose exposure history is uncertain. Interpreting these results remains challenging, as sensitization does not equate to clinical allergy, and unnecessary dietary avoidance can have nutritional, psychological, and immunological consequences [4].

Recent EAACI guidelines underline that although oral food challenge (OFC) remains the diagnostic reference standard, in vitro tools such as sIgE to Ara h 2 and basophil activation tests (BAT) may enhance specificity and reduce the need for OFC in selected cases, particularly in children without a reliable ingestion history [1,5,6]. Importantly, current recommendations highlight the clinical value of highly specific rule-in strategies, which allow confident diagnosis in sensitized children without requiring provocation.

This raises a critical question: can peanut allergy be predicted solely using in vitro markers when clinical history is unavailable? This study addresses a clinically underexplored real-life scenario by evaluating an OFC-sparing, high-specificity in vitro diagnostic pathway in children without a reliable peanut exposure history. In this single-center study, we assess whether a unified diagnostic decision pathway based exclusively on in vitro parameters can accurately identify peanut allergy in a pediatric cohort with heterogeneous and often incomplete exposure histories, including children with absent, uncertain, or remote peanut exposure.

Several studies from Poland have described peanut sensitization patterns and molecular IgE profiles in pediatric populations, including large cohort analyses using component-resolved diagnostics [7,8,9]. These studies provide important epidemiological insights into sensitization prevalence and regional molecular patterns. However, they primarily focus on sensitization profiles or OFC-based correlations and do not specifically address diagnostic decision-making in children without a reliable peanut exposure history.

To date, no Polish study has systematically evaluated a high-specificity, OFC-sparing in vitro diagnostic decision pathway tailored to real-life clinical scenarios characterized by absent, uncertain, or remote peanut exposure. Notably, differences in cultural dietary practices, such as the frequency, form, and age of peanut consumption, as well as variations in exposure to airborne allergens, may give rise to a peanut sensitization profile that differs from those observed in other populations.

Accordingly, the present study was designed to reflect routine clinical practice and was not restricted exclusively to peanut-naïve children, but rather included a heterogeneous population with absent, uncertain, or remote exposure histories.

## 2. Materials and Methods

### 2.1. Study Population

A total of 80 children were enrolled in this prospective single-center study conducted at a tertiary pediatric allergy department between March 2018 and December 2020. Of these, 65 children aged 1–18 years were found to be sensitized to peanut, defined as serum sIgE to peanut ≥0.1 kUA/L measured within six weeks prior to inclusion. Sensitization was identified during routine screening for atopic comorbidities, during evaluation before anticipated peanut introduction, or following parental concern about possible food allergy. These 65 sensitized children formed the initial study cohort and were subsequently classified into children with peanut allergy (PA) and peanut-sensitized but tolerant (PS) groups based on clinical assessment and OFCs.

**Figure 1:**
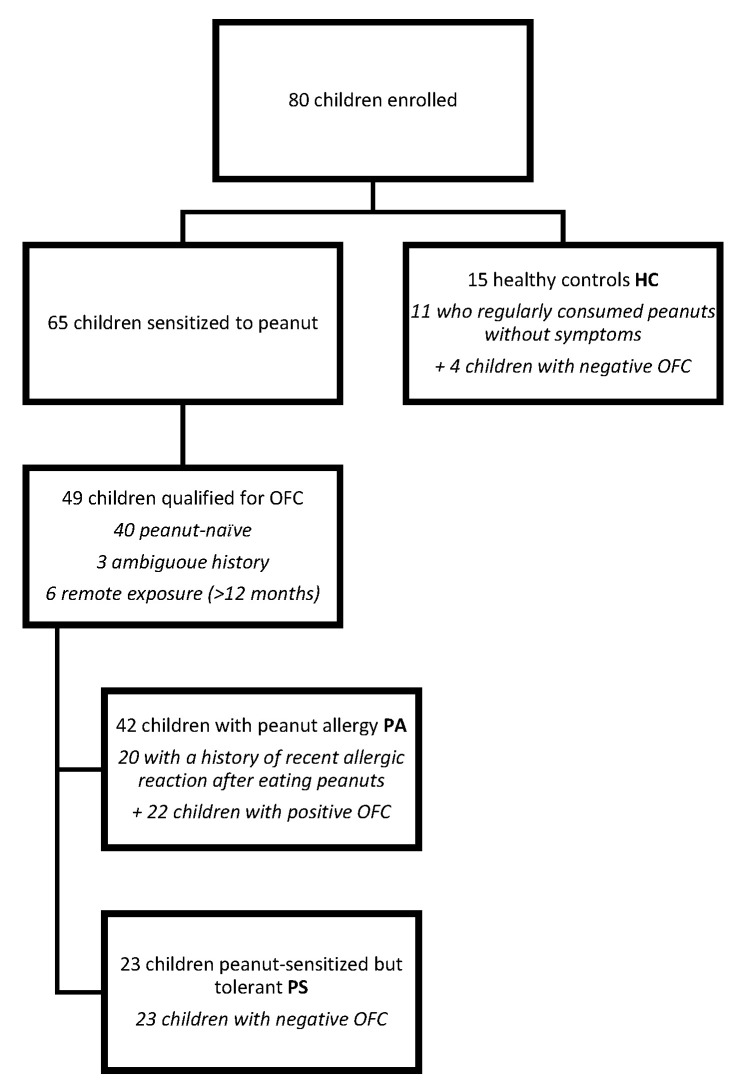
Flow of participants through the study, including enrollment of 80 children, identification of 65 peanut-sensitized participants, subsequent classification into peanut allergy (PA) and peanut-sensitized but tolerant (PS) groups, and inclusion of 15 healthy controls (HC).

The healthy control (HC) group comprised 15 age- and sex-matched children recruited from the same outpatient clinic, presenting with non-allergic complaints (e.g., abdominal pain, growth delay) and showing no history of allergic symptoms and no peanut sensitization (sIgE < 0.1 kUA/L). None had personal or parental suspicion of PA. This group served as a non-sensitized negative control, primarily for BAT comparison and background reactivity assessment. Exclusion criteria for all participants included immunodeficiencies, autoimmune diseases, malignancies, acute infections, and previous allergen immunotherapy. Figure 1 presents the flow of participants through the study.

Healthy controls underwent the same diagnostic procedures as sensitized children to evaluate background reactivity and to exclude false-positive results of in vitro assays, particularly BAT.

### 2.2. Clinical Assessment

A detailed clinical history was obtained from each participant and/or caregiver. This included demographic characteristics, atopic comorbidities (eczema, asthma, allergic rhinitis), family history of atopy, previous food-related reactions, and suspected allergies to other foods. Particular emphasis was placed on any symptoms consistent with PA, including timing, severity, reproducibility, and cofactors.

### 2.3. Skin Prick Testing

SPT was performed in all participants using food and inhalant allergens, including peanut, cow’s milk, egg white and yolk, wheat, soya, cod, hazelnut, walnut, grass pollen, alder, hazel, birch, mugwort, Dermatophagoides farinae, D. pteronyssinus, and cat and dog dander. Standardized extracts were obtained from Allergopharma–Nexter (Reinbek, Germany). A positive SPT was defined as a wheal at least 3 mm larger than the negative control.

### 2.4. Serum-Specific IgE Measurement

Serum sIgE to a broad panel of food and inhalant allergens, covering a panel similar to that used in SPT, was quantified using the Polycheck system (Biocheck GmbH, Münster, Germany). Sensitization was defined as sIgE ≥ 0.10 kUA/L.

### 2.5. Component-Resolved Diagnostics

CRDs were performed using ImmunoCAP 100 (Thermo Fisher Scientific, Waltham, MA, USA) to measure sIgE to Ara h 1, Ara h 2, Ara h 3, Ara h 6, Ara h 8, and Ara h 9. Sensitization was defined as sIgE ≥ 0.1 kUA/L.

### 2.6. Basophil Activation Test

Morning blood samples (7:00–9:00) were collected after overnight fasting. All medications potentially affecting basophil reactivity (including H1-antihistamines) were discontinued prior to testing. Samples were processed within four hours of collection. Serum for sIgE testing was obtained from clot activator tubes, whereas BAT was performed on whole blood collected into K_2_EDTA tubes.

The FlowCAST assay (BÜHLMANN Laboratories AG, Schönenbuch, Switzerland; Flow CAST. BAT Flow Cytometry. 2015) was used according to the manufacturer’s instructions. Basophils expressing CD63 after stimulation with a standardized peanut extract were quantified using flow cytometry (FACSCalibur, Franklin Lakes, NJ, USA). Anti-FcεRI antibody and fMLP served as positive controls. A BAT result was considered positive when >15% of basophils expressed CD63 or when the stimulation index (SI) was ≥2, as recommended by the manufacturer [10].

Non-responders were defined as samples in which basophils failed to respond adequately to the positive control (anti-FcεRI or fMLP), indicated by <5% CD63 expression or a stimulation index < 2. Such results were classified as non-interpretable and excluded from BAT-based analyses. All BAT results were interpreted by investigators blinded to the clinical diagnosis to minimize classification bias.

### 2.7. Oral Food Challenge

Eligibility for OFC was determined based on clinical history and test results. OFC was omitted when: a clear allergic reaction had occurred within the past 12 months and sIgE to Ara h 2 was >1.0 kUA/L, or when the child consumed peanut regularly without symptoms. Eligibility assessment was performed independently by two allergists.

Challenges followed an AAAAI open-label protocol [11]. Peanut powder (PB2, 46 g protein/100 g) was mixed with fruit purée or a tolerated dessert; the cumulative dose was 8 g of peanut. OFCs were stopped according to AAAAI criteria [11]. Anaphylaxis was diagnosed per EAACI guidelines and graded using the Sampson scale [11,12].

### 2.8. Diagnostic Decision Pathway

A multistage diagnostic decision pathway was developed. Cut-off values for sIgE to peanut extract and Ara h 2 were derived independently from ROC curve analyses within the study cohort.

Peanut allergy was diagnosed if all of the following criteria were met:

1. Sensitization: sIgE to peanut extract above the ROC-derived cut-off.
2. Component sensitization: sIgE to Ara h 2 above the ROC-derived cut-off.
3. Basophil activation: >15% activated basophils upon peanut stimulation or a stimulation index ≥ 2.

The results obtained from 15 healthy control (HC) children without peanut sensitization or allergy served as non-sensitized negative controls for baseline comparisons, particularly for BAT.

The reference standard for peanut allergy was a physician-confirmed diagnosis based on OFC or unequivocal clinical history.

In cases where a BAT result could not be obtained, an alternative diagnostic decision pathway was applied. The objective was to assess whether a simplified two-step in vitro strategy could achieve high diagnostic accuracy without requiring BAT.

Peanut allergy was diagnosed if both of the following criteria were met:

1. Sensitization: skin prick test (SPT) to peanut above the ROC-derived cut-off.
2. Component sensitization: sIgE to Ara h 2 above the ROC-derived cut-off.

ROC curves, sensitivity, specificity, PPV, and NPV were calculated for both diagnostic approaches. For composite diagnostic decision pathways, ROC curves and AUC values were generated using an ordinal decision score reflecting progression through successive diagnostic steps, rather than a single binary outcome.

To directly assess the incremental value of BAT, a head-to-head comparison of composite diagnostic pathways with and without BAT was performed, restricting the analysis to participants with interpretable BAT results.

The selection of variables included in the diagnostic decision pathways was based on individual test performance and biological complementarity. Although sIgE to Ara h 6 demonstrated good discriminatory ability, its diagnostic information largely overlapped with that of sIgE to Ara h 2, and its inclusion did not improve pathway performance. Similarly, sIgE to whole peanut extract showed a strong correlation with Ara h 2–specific IgE and did not provide additional independent diagnostic value beyond sIgE to Ara h 2.

ROC analyses for individual tests (SPT, sIgE to peanut extract, and component-resolved diagnostics) were performed in the entire cohort (n = 80). The performance of BAT and any BAT-dependent decision pathway steps was evaluated only in participants with interpretable BAT results, while non-interpretable BAT results were excluded from BAT-based analyses but not from ROC analyses of the other tests.

### 2.9. Ethical Considerations

The study was approved by the Local Bioethics Committee (No. KB 234/2019). Written informed consent was obtained from parents/guardians and from children aged ≥ 16 years.

### 2.10. Statistical Analysis

Categorical variables were summarized as frequencies and percentages. Continuous variables were tested for normality using the Shapiro–Wilk test and are presented as means with standard deviations or medians with interquartile ranges, as appropriate. Between-group comparisons were performed using Student’s *t*-test, the Mann–Whitney U test, or the chi-square test.

Diagnostic performance was assessed using receiver operating characteristic (ROC) curve analysis for the following individual markers: SPT to peanut extract, sIgE to peanut, and sIgE to Ara h 2, in the entire cohort of 80 children. Analyses involving BAT were restricted to participants with interpretable BAT results. Cut-off values were determined using Youden’s Index. For each test and for composite diagnostic decision pathways, the area under the curve (AUC), sensitivity, specificity, positive predictive value (PPV), and negative predictive value (NPV) were calculated, along with their corresponding 95% confidence intervals (CIs), based on the Wilson score method. For composite diagnostic decision pathways, ROC curves and AUC values were generated using an ordinal scoring system reflecting progression through successive diagnostic steps rather than a single binary outcome. Likelihood ratios (positive and negative) were also computed, and DeLong’s test was used to compare AUCs.

The multistage composite decision pathway (SPT + sIgE to peanut + sIgE to Ara h 2 ± BAT) was evaluated as a high-specificity rule-in strategy. Diagnostic accuracy and the proportion of OFCs avoided were also reported. A secondary analysis was conducted for a simplified decision pathway using only sIgE to Ara h 2 and SPT to peanut (≥4 mm) to assess whether comparable performance could be achieved without BAT. All statistical analyses were performed using IBM SPSS Statistics for Windows, version 28.0 (IBM Corp., Armonk, NY, USA), with *p* < 0.05 considered statistically significant.

## 3. Results

### 3.1. Patient Group Allocation

Based on sensitization status, clinical symptoms, and the results of the OFCs, children were assigned to diagnostic groups as shown in Figure 1. The characteristics of the patients are presented in Table 1.

### 3.2. History of Peanut Exposure and Symptoms

Among the entire cohort, 40 children (50%) had a documented history of peanut consumption at some point in their lives. Of these: 26 children (65%) experienced unequivocal symptoms consistent with PA, 3 children (7.5%) reported ambiguous or non-specific symptoms and 11 children (27.5%) remained asymptomatic. For the remaining 40 children (50%), parents were unable to confirm whether peanut ingestion had ever occurred.

The mean age at onset of the first allergic symptoms was 2.5 ± 0.9 years (range 12 months–4 years; median 2 years). Among children who had ever consumed peanut, cutaneous manifestations such as urticaria, erythema, and pruritus were the most frequently reported symptoms (25 children; 96.2%). Respiratory symptoms (mainly cough) occurred in 17 children (65.4%), while gastrointestinal complaints, including diarrhea, were reported in 12 children (46.2%). Abdominal pain and vomiting occurred in 8 children (30.8%). Additionally, 12 children (52.2%) exhibited anxiety or behavioural changes during or after peanut exposure. Syncope was reported in 6 children (23.1%). Regarding treatment, antihistamines were the most commonly administered therapy (21 children; 80.8%), while epinephrine was required in 10 cases (38.5%).

### 3.3. Sensitization Assessment: SPT, sIgE, and BAT

The results of SPT, serum sIgE, and BAT are summarized in Table 2 for the PA and PS groups. These tests were also performed in the HC group, yielding consistently negative results.

**Figure 2:**
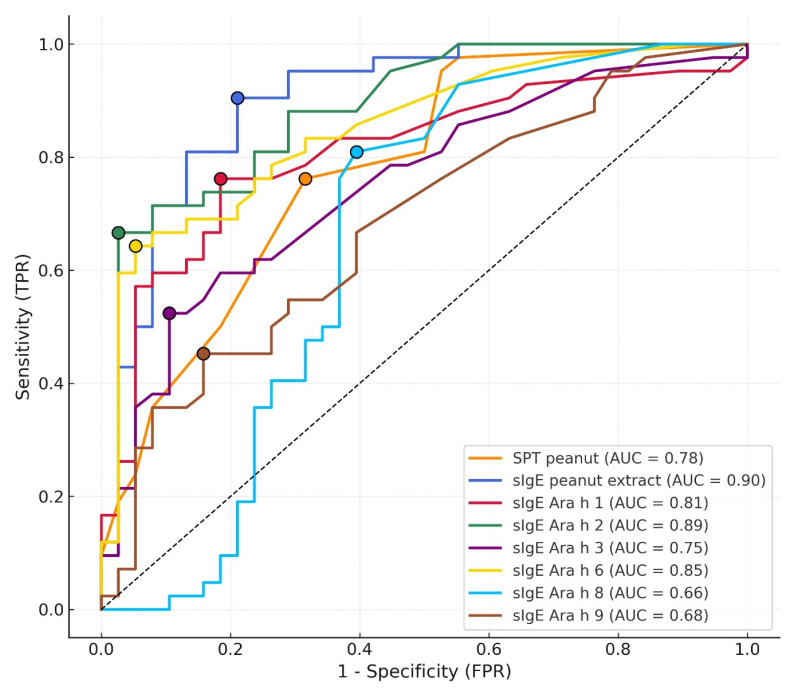
ROC curves for SPT, sIgE to peanut extract, and peanut component-specific sIgE (Ara h 1, Ara h 2, Ara h 3, Ara h 6, Ara h 8, Ara h 9). AUC values are shown in the legend. Optimal diagnostic thresholds were defined using the Youden index: sIgE peanut extract ≥ 3.21 kUA/L, sIgE Ara h 1 ≥ 0.25 kUA/L, sIgE Ara h 2 ≥ 2.64 kUA/L, sIgE Ara h 3 ≥ 0.37 kUA/L, sIgE Ara h 6 ≥ 1.09 kUA/L, sIgE Ara h 8 ≥ 0.04 kUA/L, sIgE Ara h 9 ≥ 0.23 kUA/L, SPT peanut ≥ 4.0 mm.

BAT results were considered non-interpretable and excluded from BAT-based analyses if predefined validity criteria were not met, including either inadequate basophil response to positive controls or failure to meet the stimulation index (SI) requirement. Among the 80 children, 21 (26.3%) had non-interpretable BAT results, including 16 non-responders and 5 samples with insufficient stimulation index, and were excluded from BAT-based analyses. In diagnostic accuracy assessment, sIgE to peanut extract and Ara h 2 achieved the highest AUC values (0.90 and 0.89), with Ara h 6 (0.85), Ara h 1 (0.81) and SPT (0.78) showing moderate performance, while Ara h 3, Ara h 8 and Ara h 9 demonstrated limited discriminatory capacity (Figure 2).

### 3.4. Oral Food Challenge Outcomes

Of the 80 enrolled children, 49 (61.3%) were qualified for OFC according to the study protocol. A total of 49/80 children (61.3%) underwent OFC. In 31 children (38.8%), OFC was not performed: 11 (13.8%) regularly consumed peanuts without symptoms (classified as HC), and 20 (25.0%) had a clear allergic reaction within the previous 12 months together with Ara h 2 >1.0 kUA/L (classified as PA). Among the 49 children who underwent OFC, 40 (81.6%) were peanut-naïve, 3 (6.1%) had previously ingested peanut with inconclusive symptoms, and 6 (12.2%) had a remote history of peanut-allergic reactions (>12 months). OFC results were negative in 27 (55%) and positive in 22 (45%) children. The final allocation of participants to the subgroups is shown in Figure 1. During OFC, the most frequent symptoms were urticaria (20 children, 40.8%) and pruritus: 19 children (38.8%). A detailed breakdown is provided in Table 3.

### 3.5. Performance of the Diagnostic Decision Pathway

**Figure 3:**
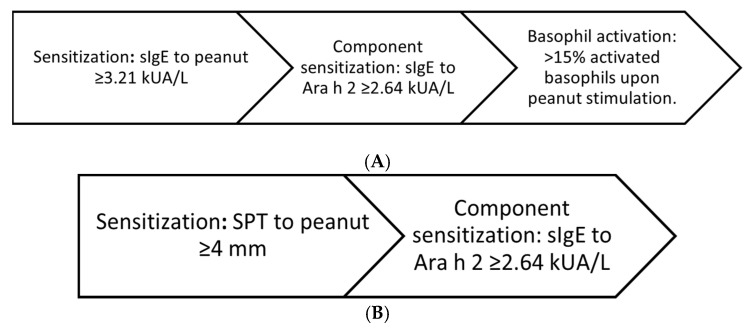
(**A**). Schematic representation of the multistage diagnostic decision pathway for peanut allergy. The algorithm integrates stepwise assessment of sIgE to peanut extract (≥3.21 kUA/L), sIgE to Ara h 2 (≥2.64 kUA/L), and basophil activation test (BAT) positivity defined as >15% activated basophils upon peanut stimulation. The algorithm was constructed using markers providing complementary diagnostic information, while highly correlated serological components were intentionally not combined. (**B**). Schematic representation of the alternative two-step diagnostic decision pathway for peanut allergy. The algorithm integrates stepwise assessment of skin prick test (SPT) to peanut extract (≥4 mm) and sIgE to Ara h 2 (≥2.64 kUA/L). The algorithm was constructed using markers providing complementary diagnostic information, while highly correlated serological components were intentionally not combined.

A multistage diagnostic decision pathway integrating SPT, sIgE to peanut, sIgE to Ara h 2, and BAT was evaluated for its ability to identify PA with high specificity and OFC-sparing potential (Figure 3A). The decision pathway achieved a specificity of 100%, with a PPV of 100%, allowing clinicians to avoid OFC in 28.6% of sensitized children. However, its sensitivity remained moderate (~58.5%), limiting its rule-out capacity. ROC analysis yielded an AUC of approximately 0.75 (95% CI ≈ 0.60–0.85), with a negative likelihood ratio of ~0.42, consistent with a decision pathway optimized for confirmation rather than exclusion.

In a secondary analysis, a simplified two-step decision pathway using only sIgE to Ara h 2 (≥2.64 kUA/L) and SPT to peanut (≥4 mm) was assessed to determine whether high diagnostic performance could be preserved without BAT. This decision pathway yielded a specificity of 100%, sensitivity of 53.7%, and would have avoided OFC in 27.5% of cases (Figure 3B). While the simplified decision pathway demonstrated slightly reduced PPV compared to the full algorithm, it preserved clinical utility in settings where BAT is unavailable. Performance characteristics of diagnostic strategies: sIgE to peanut extract, sIgE to Ara h 2, and both decision pathways is presented in Table 4. Because BAT was not interpretable in all participants, performance metrics in Table 4 reflect the decision pathway applied to the entire cohort (n = 80), whereas a head-to-head comparison restricted to participants with interpretable BAT results (n = 71) is provided separately in Appendix A Table A1.

## 4. Discussion

In this preliminary study, three main observations emerged. First, sIgE to peanut and Ara h 2 remained the most informative stand-alone markers in children with uncertain ingestion history. Second, the unified multistage decision pathway achieved 100% specificity, establishing a high-certainty diagnostic zone that may reduce reliance on OFC. Third, the substantial number of BAT non-responders limits its feasibility as a mandatory component of such algorithms, positioning BAT primarily as a confirmatory test when feasible. Importantly, the novelty of the present study lies not in the identification of new biomarkers, but in the real-life application of a high-specificity, OFC-sparing in vitro decision pathway tailored to children with uncertain or absent peanut exposure history.

This study examined whether an exclusively in vitro diagnostic pathway can support clinical decision-making in children with uncertain or absent peanut ingestion history. The diagnostic challenge in this population lies in distinguishing clinically relevant allergy from asymptomatic sensitization, and our findings suggest that a structured, multistage laboratory strategy may be clinically valuable in selected scenarios where OFC is undesirable, delayed, or not feasible.

In our cohort, sIgE to peanut extract and sIgE to Ara h 2 demonstrated strong individual discriminatory performance (AUC 0.895 and 0.889), consistent with extensive evidence identifying sIgE to Ara h 2 as the most clinically informative serological marker for pediatric PA [1]. The cut-off values observed in our cohort align with those reported in other populations, despite differences in dietary habits and geographic latitude. Studies by Klemans et al. confirm that sIgE to Ara h 2 offers superior specificity and diagnostic accuracy compared with whole-extract sIgE, especially in children lacking documented ingestion [13]. Multi-omics approaches similarly highlight sIgE to Ara h 2 as a key biomarker closely linked to clinical reactivity [14]. Nevertheless, elevated sIgE to Ara h 2 levels do not invariably predict allergy, nor do low levels reliably exclude it, emphasizing the limitations of single-analyte serology. Continuous likelihood-ratio modelling, as proposed by Nalin et al., reinforces the need for nuanced interpretation of sIgE rather than binary thresholding [15].

Several studies from Poland have provided valuable data on peanut sensitization prevalence and molecular IgE profiles in pediatric populations, including analyses using component-resolved diagnostics and correlations with OFC outcomes [7,8,9]. These studies have substantially contributed to understanding regional sensitization patterns. However, they primarily focused on sensitization profiles rather than diagnostic decision-making in children without reliable peanut exposure history. In contrast, the present study addresses this specific real-life clinical gap by evaluating a structured, high-specificity in vitro diagnostic decision pathway aimed at avoidance of unnecessary OFC in definitively allergic patients.

In this context, our multistage decision pathway achieved 100% specificity and PPV, defining a high-certainty rule-in zone in which OFC can be omitted in a high-certainty diagnostic setting. These findings align with external evidence: Brettig et al. demonstrated that combining SPT with sIgE to Ara h 2 reduces OFC use by ~28% and lowers diagnostic costs by ~32% [16]. BSACI and EAACI guidelines also acknowledge that high sIgE to Ara h 2 values may obviate the need for OFC when consistent with clinical context [1,17]. The structure of the proposed diagnostic pathways reflects the strong correlation between sIgE to Ara h 2, Ara h 6, and whole peanut extract, which limits the incremental value of combining multiple serological markers. In this context, SPT was retained in the simplified model as the only test providing an independent functional readout capable of modifying the diagnostic interpretation of sIgE results when BAT was unavailable or non-interpretable.

When interpretable, BAT contributed to the decision pathway’s specificity. However, given its limited feasibility, high technical demands, and >25% rate of non-interpretable BAT results in our cohort, BAT should be considered an optional, second-line tool rather than an essential component. In clinical practice, the multistage algorithm may need to rely on sIgE to Ara h 2 and SPT alone in settings where BAT is unavailable or yields non-interpretable results. Notably, allergic patients not identified by the rule-in pathways generally showed less distinct laboratory profiles; however, no conclusions regarding reaction severity can be drawn, as current diagnostic tests do not reliably predict clinical severity.

It should be acknowledged that alternative diagnostic strategies could be derived from the present data, including approaches based on dual cut-off values for sIgE to Ara h 2 alone, allowing both rule-in and rule-out classification with OFC reserved for intermediate values. Indeed, such strategies may further reduce the number of required OFCs at the cost of accepting a small number of false-positive or false-negative classifications. However, the present study deliberately focused on a high-specificity rule-in approach, prioritizing diagnostic certainty and minimization of false-positive diagnoses in children without a reliable peanut ingestion history, for whom an incorrect diagnosis of peanut allergy may result in long-term and unnecessary dietary restriction. These alternative strategies are therefore considered complementary and warrant evaluation in future, prospectively designed studies.

In our exploratory analysis, a simplified two-step decision pathway combining sIgE to Ara h 2 and SPT to peanut extract showed promising discriminatory performance. Using defined cut-offs (sIgE Ara h 2 ≥ 2.64 kUA/L and SPT ≥ 4 mm), this approach achieved 100% specificity and avoided the need for OFC in approximately 27.5% of children while maintaining a high PPV. Although slightly less sensitive than the full decision pathway, it represents a pragmatic alternative when BAT is unavailable, as it uses widely accessible tests. Similar simplified decision pathways have been reported by Brettig et al. and endorsed by BSACI and EAACI guidelines [1,16,17].

A notable proportion of non-responders (20%) failed to activate even with positive control stimulation, with an additional 6% not fulfilling stimulation index thresholds. This observation is consistent with previous reports demonstrating limited BAT interpretability in pediatric populations, potentially related to age-dependent basophil reactivity, FcεRI expression, or technical variability [18,19]. Future studies should stratify BAT performance by these modifiers and consider parallel testing with alternative markers.

These findings are increasingly relevant in the context of rising test utilization, expanded sensitization screening after LEAP, and the documented increase in PA diagnoses [20,21]. Sensitization without clinical allergy often leads to unnecessary dietary restriction, psychological burden, and reduced quality of life, underscoring the importance of diagnostic strategies that reinforce certainty and prevent overdiagnosis [22]. The proposed decision pathway can assist clinicians and families in navigating decisions about home introduction versus supervised challenge, particularly in emotionally complex situations or in settings with limited OFC capacity.

Despite its strengths, any serological test cannot replace OFC in all clinical contexts. OFC remains the definitive method for assessing clinical reactivity and continues to serve as the diagnostic reference standard. Our results, however, align with European pediatric studies demonstrating the strong performance of Ara h 2 and the limited feasibility of BAT in routine practice [1]. The present analysis adds to this evidence by showing that a highly specific, multistage laboratory decision pathway complements single-marker serology and provides an OFC-sparing pathway for a clinically relevant subgroup of sensitized children without documented peanut exposure. Clinicians encountering uninterpretable BAT results must revert to the simplified two-tier approach (sIgE + SPT) or proceed with OFC, highlighting the necessity of flexibility in applying such decision pathways.

Taken together, these findings support the hypothesis that a fully in vitro multistage decision pathway can support reliable distinction between PA and tolerance in this diagnostically challenging population. Future prospective studies should evaluate simplified in vitro decision pathways without BAT and determine whether Ara h 2–based thresholds alone can achieve similar OFC-sparing effects.

This study has several limitations. First, the relatively small sample size, particularly within the core sensitized cohort (n = 65), limits statistical power and may affect the precision of the derived cut-off values. Second, the single-center design may restrict the generalizability of the findings to other populations with different referral patterns, dietary habits, or sensitization profiles. Third, although the cohort included children across a wide age range (1–18 years), the study was not powered to perform age-stratified analyses of diagnostic thresholds or pathway performance. Unlike cow’s milk or hen’s egg allergy, no universally accepted age-specific diagnostic cut-offs have been established for peanut allergy, particularly for Ara h 2–specific IgE; therefore, age-independent thresholds were applied in this real-life pediatric cohort. Finally, the proposed diagnostic decision pathway should be regarded as a derivation-phase approach and requires external validation in larger, independent, multicenter cohorts before broader clinical implementation.

## 5. Conclusions

A fully in vitro diagnostic strategy incorporating sIgE to peanut, sIgE to Ara h 2, and BAT can provide a high-certainty, OFC-sparing pathway for selected sensitized children without a reliable ingestion history. While sIgE to Ara h 2 remains the most efficient first-line discriminator, the multistage decision pathways offer added clinical value by defining a high-specificity diagnostic allowing definitively allergic patients to avoid an unnecessary and potentially risky OFC. Broader validation is required, but these findings support the integration of structured laboratory-based algorithms into individualized diagnostic decision-making for pediatric PA.

## Figures and Tables

**Table 1:** Characteristics of the study group.

Parameter	Children with Peanut Allergy PA n = 42 (100%)	Children with Peanut Sensitization PS n = 23 (61%)	Healthy Children HC n = 15 (100%)	*p* *	*p* **
Age, years					
Mean ± SD	5.93 ± 3.13	5.39 ± 3.39	4.07 ± 2.28	0.53	0.16
Min–max	1–15	2–16	1–10		
Sex, n (%)					
Female	9 (21.43)	7 (30.43)	7 (46.67)	0.72	0.49
Male	33 (78.57)	16 (69.56)	8 (53.33)		
Place of living, n (%)					
Village	14 (33.33)	18 (78.26)	8 (53.33)	0.09	0.16
City	28 (66.67)	5 (21.74)	7 (46.67)		
Concomitant diseases					
Atopic dermatitis, n (%)	39 (92.86)	19 (82.61)	0	0.53	<0.01
Asthma, n (%)	24 (57.14)	8 (21.05)	0	0.19	0.01
Allergic rhinitis, n (%)	20 (47.62)	15 (65.22)	0	1.00	0.21
Anaphylaxis, n (%)	36 (85.71)	8 (34.78)	0	<0.01	<0.01
Other food allergy, n (%)	35 (83.33)	18 (78.26)	0	0.88	<0.01
Atopy in family					
One parent, n (%)	18 (42.86)	10 (43.49)	4 (26.6)	1	0.36
Both parents, n (%)	8 (19.05)	4 (17.39)	2 (13.33)	0.99	1
Siblings, n (%)	10 (23.81)	8 (34.78)	3 (20)	0.64	1

*p* * PA vs. PS; *p* ** PA vs. HC.

**Table 2:** Comparison of SPT, serum sIgE, CRD and BAT results in children with peanut allergy (PA) and peanut sensitization without allergy (PS).

Parameter	PA n = 42 (100%)	PS n = 23 (61%)	*P*
SPT wheal, mm, mean ± SD	4.88 ± 2.35	3.52 ± 2.09	0.02
sIgE peanut extract, kUA/L, mean ± SD	26.84 ± 20.48	7.51 ± 19.81	<0.01
sIgE Ara h 1, kUA/L, mean ± SD	10.87 ± 25.56	0.65 ± 1.67	0.02
sIgE Ara h 2, kUA/L, mean ± SD	20.58 ± 30.21	2.76 ± 11.51	<0.01
sIgE Ara h 3, kUA/L, mean ± SD	5.22 ± 15.87	0.57 ± 1.57	0.07
sIgE Ara h 6, kUA/L, mean ± SD	16.53 ± 27.91	2.37 ± 10.19	<0.01
sIgE Ara h 8, kUA/L, mean ± SD	4.04 ± 7.7	17.67 ± 31.14	0.55
sIgE Ara h 9, kUA/L, mean ± SD	4.23 ± 7.7	2.31.67 ± 9.23	0.55
Unstimulated (background) %, mean ± SD	1.43 ± 1.67	1.76 ± 1.50	0.56
Stimulated FCεRI %, mean ± SD	67.78 ± 27.96	65.90 ± 26.9	0.85
Stimulated f-MLP %, mean ± SD	28.96 ± 18.60	31.26 ± 24.72	0.78
Stimulated peanut extract %, mean ± SD	39.88 ± 32.66	6.14 ± 5.76	<0.01

**Table 3:** Clinical symptoms observed in children with positive oral food challenges.

Symptoms	Children with Positive OFC n = 22 (100%)
Subjective symptoms. n (%)	
Itching Itchy tongue or throat/numbness of tongue and lips Shortness of breath Stomach pain Anxiety/drowsiness/change in behavior	19 (86.36) 16 (72.73) 6 (27.27) 5 (22.73) 17 (77.27)
Objective symptoms. n (%)	
Urticaria Erythematous rash Angioedema Rhinitis Stridor Persistent cough Wheezing Vomiting Diarrhea Hypotension	20 (90.91) 4 (18.18) 15 (68.18) 12 (54.54) 3 (13.64) 11 (50) 7 (31.82) 9 (40.91) 6 (27.27) 3 (13.64)

Anaphylaxis was diagnosed in 11 children (22.4%). Severity grading: grade I in 3 children (27.3%), grade II in 4 children (36.4%), and grade III in 4 children (36.4%). The mean cumulative tolerated dose was 902.5 ± 1385.1 mg of peanut protein. The mean eliciting dose was 856.0 ± 511.2 mg.

**Table 4:** Performance characteristics of three diagnostic strategies: sIgE to peanut extract, sIgE to Ara h 2, and both decision pathways *.

Test (Cut-Off)	AUC (95% CI)	Sensitivity ** (95% CI)	Specificity ** (95% CI)	PPV ** (95% CI)	NPV ** (95% CI)	LR^+^ (95% CI)	LR^−^ (95% CI)	OFC Avoided (%)
sIgE Peanut (≥3.21 kUA/L)	0.895 (0.814–0.958)	90.2% (76.9–97.3%)	76.9% (60.7–88.9%)	80.4% (66.1–90.6%)	88.2% (72.5–96.7%)	3.9 (2.4–8.6)	0.13 (0.03–0.27)	–
sIgE Ara h 2 (≥2.64 kUA/L)	0.889 (0.810–0.951)	68.3% (51.9–81.9%)	97.4% (86.5–99.9%)	96.6% (82.2–99.9%)	74.5% (60.4–85.7%)	26.7 (7.1–∞)	0.32 (0.18–0.48)	–
SPT Peanut (≥4 mm)	0.70 (0.60–0.80)	78.0% (62–89%)	69.2% (52–83%)	72.7% (57–85%)	75.0% (58–88%)	2.5 (2.1–7.3)	0.32 (0.18–0.46)	-
Composite Decision Pathway # (sIgE Peanut + Ara h 2 ± BAT)	0.75 (0.60–0.85)	~58.5% (42.1–73.7%)	100% (91.0–100%)	100% (85.8–100%)	~69.6% (55.9–81.2%)	∞ (≫10)	~0.42 (0.26–0.58)	28.6%
Composite Decision Pathway (SPT Peanut ≥ 4 mm + sIgE Ara h 2 ≥ 2.64)	0.77 (0.68–0.86)	53.7% (0.41–0.70%)	100.0% (91.0–100%)	100.0% (86–100%)	67.2% (56–79%)	∞ (≫10)	0.46 (0.28–0.48)	27.5%

* Ara h 6 and additional peanut components were not included in the composite decision pathways due to strong correlation with Ara h 2 and lack of incremental diagnostic value. ** Sensitivity, specificity, PPV, and NPV are reported as percentage estimates with confidence intervals; apparent discrepancies when back-calculating absolute patient numbers are due to rounding and statistical estimation. # The notation “±BAT” indicates that BAT constituted the third confirmatory step when interpretable; when BAT was unavailable or non-interpretable, the predefined alternative BAT-free pathway was applied.

## Data Availability

The data supporting the findings of this study are available from the corresponding authors upon reasonable request. Due to ethical and privacy restrictions, the datasets are not publicly accessible.

## Abbreviations

The following abbreviations are used in this manuscript:

PA	Peanut Allergy
LEAP	Learning Early About Peanut Allergy
CRD	Component-Resolved Diagnostics
SPT	Skin Prick Test
EAACI	European Academy of Allergy and Clinical Immunology
OFC	Oral Food Challenge
BAT	Basophil Activation Test
HC	Healthy Controls
PS	Peanut Sensitized
EDTA	Ethylenediaminetetraacetic Acid
AAAAI	American Academy of Allergy, Asthma and Immunology
ROC	Receiver Operating Characteristic
PPV	Positive Predictive Value
NPV	Negative Predictive Value
AUC	Area Under the Curve
LR+	Positive Likelihood Ratio
LR-	Negative Likelihood Ratio

## Appendix A

A head-to-head comparison of the composite diagnostic pathways incorporating sIgE to peanut extract and Ara h 2, with and without the inclusion of BAT, was performed restricting the analysis to participants with interpretable BAT results (n = 71) (Table 1). Both approaches achieved 100% specificity and PPV. The inclusion of BAT did not improve specificity but resulted in a reduction in sensitivity compared with the BAT-free pathway. These findings support the role of BAT as an optional confirmatory test rather than a mandatory component of the diagnostic decision pathway.

**Table A1:** Head-to-head comparison of composite diagnostic pathways with and without BAT restricted to participants with interpretable BAT results (n = 71).

Test (Cut-off)	AUC (95% CI)	Sensitivity ** (95% CI)	Specificity ** (95% CI)	PPV ** (95% CI)	NPV ** (95% CI)	LR^+^ (95% CI)	LR^−^ (95% CI)	OFC Avoided (%)
Composite Decision Pathway (sIgE Peanut + Ara h 2)	0.905 (0.834–0.961)	~65.0% (49.5–77.9%)	100% (89.0–100%)	100% (87.1–100%)	~68.9% (54.3–80.5%)	∞ (≫10)	~0.35 (0.24–0.54)	42.6%
Composite Decision Pathway (sIgE Peanut + Ara h 2 ± BAT)	0.905 (0.832–0.962)	~57.5% (42.2–71.5%)	100% (89.0–100%)	100% (85.7–100%)	~64.6% (50.4–76.6%)	∞ (≫10)	~0.43 (0.30–0.62)	37.7%

** Sensitivity, specificity, PPV, and NPV are reported as percentage estimates with confidence intervals; apparent discrepancies when back-calculating absolute patient numbers are due to rounding and statistical estimation.
